# Second generation tyrosine kinase inhibitors for the treatment of metastatic non-small-cell lung cancer

**DOI:** 10.1186/2213-0802-2-2

**Published:** 2014-01-06

**Authors:** Irene Stasi, Federico Cappuzzo

**Affiliations:** Medical Oncology Department, IstitutoToscanoTumori, OspedaleCivile, Livorno, Italy

**Keywords:** Afatinib, Dacomitinib, Erlotinib, Neratinib, CO-1686, AP26113, BMS-690514, XL647 EGFR, NSCLC

## Abstract

**Backgruond:**

Since their first description, activating epidermal growth factor receptor (*EGFR*) mutations identify a distinct clinical entity of patients with non-small-cell lung cancer (NSCLC).

**Findings:**

New targeted therapies for molecularly selected NSCLC are changing the natural history of the disease, with results superior to standard chemotherapy as demonstrated in large phase III studies with first generation EGFR tyrosine kinase inhibitors (TKIs) erlotinib and gefitinib. However, after an initial response, all patients inevitably progress and several mechanisms including a secondary mutation in exon 20 of the *EGFR* gene (T790M) or *MET* or *HER2* amplifications are responsible for acquired resistance (AR). In clinical practice few options are available for patients with AR, and several new agents or strategies are currently under investigation, including second generation TKIs.

**Conclusions:**

Aim of the present review is to present available data on new EGFR-TKIs and to discuss how these agents could overcome AR to erlotinib or gefitinib.

## Findings

In the last few years, the treatment of advanced non-small-cell lung cancer (NSCLC) has radically changed, due to advances in cancer biology. The old-fashioned ‘one size fits all’ chemotherapeutic approach is nowadays replaced by a careful selection mainly based on tumor histology and, most importantly, on biological characteristics. Specifically, the discovery of the biologic and therapeutic importance of acquired genetic alterations in two genes that encode pharmacologically targetable tyrosine kinases, the Epidermal Growth Factor Receptor (*EGFR*) and Anaplastic Lymphoma Kinase (*ALK*) has changed the way these cancers are treated.

In 2004, *EGFR* gene mutations were firstly identified: classical-activating *EGFR* mutations are localized in exon 19, mainly consisting of an in-frame deletion (45-50%), and in exon 21, consisting of the L858R point mutation (40-45%), even if there are less common mutations localized in other exons [1–3]. Since their identification, it was clear that *EGFR* mutations, more frequently observed in never smokers, adenocarcinoma histology, women and Asiatic patients, outline a distinct subgroup of NSCLC. During the last years, six phase III trials (Table 1) established that patients harboring activating *EGFR* mutations benefit more from a first line treatment with an EGFR tyrosine kinase inhibitor (TKI), such as erlotinib or gefitinib, than from standard chemotherapy, at least in terms of response rate (RR), progression-free survival (PFS) and quality of life [4–9]. On the basis of these solid results, regulatory agencies have progressively approved EGFR-TKIs for the first line treatment of NSCLC harbouring activating *EGFR* mutations.

**Table 1:** **First line trials of 1** ^**st**^ **generation TKI in EGFR mutated patients**

Study	N	OR%	mPFS (mo)	mOS (mo)
**First Signal** ^**4**^				
Gefitinib	26	85	8	27.2
Cis/Gem	16	38	2.1	25.6
**IPASS** ^**5**^				
Gefitinib	132	71	9.5	19
Carbo/Taxol	129	47	6.5	18
**WJTOG** ^**6**^				
Gefitinib	86	62	9.2	30.9
Cis/Doce	86	31	6.3	NR
**NEJOG** ^**7**^				
Gefitinib	114	74	10.8	27.7
Carbo/Taxol	110	31	5.4	26.6
**OPTIMAL** ^**8**^				
Erlotinib	83	83	13.1	NR
Carbo/Gem	72	36	4.6	NR
**EURTAC** ^**9**^				
Erlotinib	86	58	9.7	NR
Plat Doublet	87	15	5.2	NR

*N*, number of patients; *OR*, objective response rate; *mPFS*, median progression free servival; mOS, median overall survival; *mo*, months; *cis*, cisplatin; gem, gemcitabine; *carbo*, carboplatin; doce, docetaxel; plat, platinum; *NR*, not reached.

**Figure 1:**
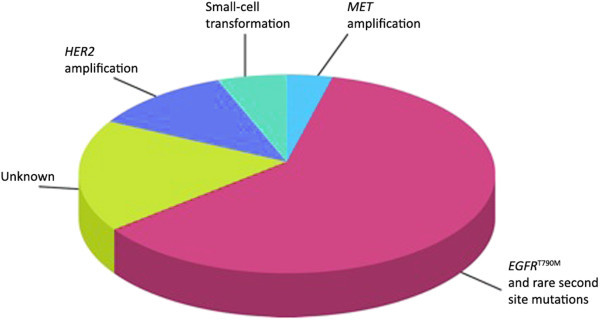
**Mechanisms responsible for acquired resistance (adapted from** http://cancerdiscovery.aacrjournals.org **).**

Unfortunately, after a median of 8–10 months, all responding patients develop acquired resistance (AR) to EGFR-TKI therapy, with the inevitable consequence of disease progression. Recent studies demonstrated that several mechanisms are responsible for AR (Figure 1), with approximately 30% of patients in which resistance factors are not yet identified [10]. The firstly described and the most common event responsible for resistance is the acquisition of the T790M missense mutation, which is found in ≈ 50% of patients progressing after an initial response to erlotinib or gefitinib [11, 12]. Other less frequent mechanisms include secondary mutations within *EGFR*[13, 14], *MET* amplification [15], *HER2* amplification [16, 17], small cell histologic transformation [18]. Identification of mechanisms responsible for AR has therapeutic implications, and several agents are currently under investigation particularly for individuals with the secondary T790M mutation.

The family of 2^nd^ generation TKIs exploit three basic approaches aimed at increasing the efficacy over first-generation EGFR-TKIs and overcoming the AR, such as intensification of EGFR inhibition, targeting a specific alteration of the EGFR downstreaming signaling and finally combining the EGFR plus alternate pathway inhibition. This review will discuss the mechanism of action, the available data and the future implications of second-generation EGFR-TKIs for the treatment of advanced NSCLC.

### Intensification of EGFR inhibition

**Figure 2:**
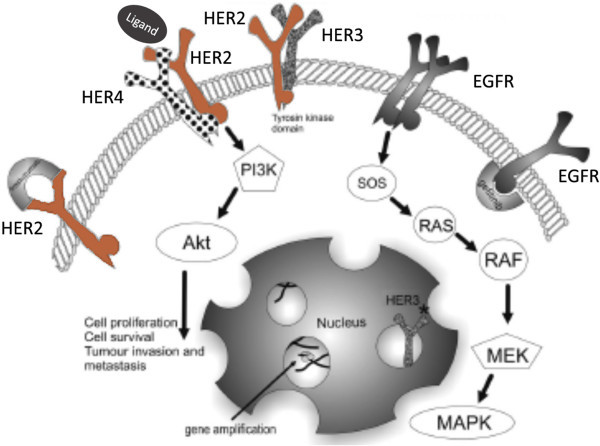
**Epidermal Growth Factor Receptor Family and intracellular pathway (adapted from** http://nature.com **).**

*EGFR* mutations identify a distinct subgroup of NSCLC characterized by oncogene addiction, for which cell’s growth and survival signals are dependent upon EGFR activation. In this scenario, cells would develop resistance mechanisms that reactivate EGFR despite the presence of an inhibitor, as the acquired T790M second site mutation in the exon 20 of EGFR gene. The T790M missense mutation could be classified as the gatekeeper mutation, occurring within the ATP-binding site and interfering with the first-generation TKI’s binding by steric hindrance, causing a bulky methionine side chain in the receptor kinase domain [19]. In vitro studies showed that exposing *EGFR*-mutant lung cancer cell lines to a mutagen and culturing them in the presence of an EGFR-TKI, the resistant clones with the T790M mutation maintained a persistent phosphorylation [20]. Given this role of persistent *EGFR* signaling, many trials evaluated the intensification of EGFR inhibition through the use of drug molecules with additional activity against other receptors in the EGFR family, as the second-generation neratinib, dacomitinib and afatinib [21]. These inhibitors are mainly different from erlotinib and gefitinib for two features: each forms a covalent and irreversible attachment to the EGFR kinase domain, and each also inhibits other members of the ERBB family (Figure 2).

### Neratinib

Neratinib is an oral, irreversible inhibitor of both EGFR and HER2; in preclinical studies conducted on cell lines with both an activating *EGFR* mutation and the *T790M*, neratinib was more effective at suppressing cell proliferation than gefitinib [22]. After a phase I trial, recruiting heavily pretreated patients with NSCLC in which the maximum tolerated dose (MTD) was at 320 mg daily [23], neratinib was studied in a phase II study. During the study, due to the development of grade 3 diarrhea in more than 50% of patients at MTD, the dose was decreased to 240 mg orally daily. Unfortunately the drug showed modest clinical efficacy with only 3% response rate in 91 *EGFR* mutant patients included in a phase II study and with no response in patients EGFR T790M mutated [24]. The lack of efficacy could probably be related to the high concentrations of neratinib required in preclinical studies to inhibit EGFR T790M and the limitations of clinical dosing.

### Dacomitinib

Dacomitinib is an irreversible EGFR, HER2 and HER4 inhibitor with a higher kinase inhibition than gefitinib/erlotinib in both gefitinib/erlotinib-sensitive and in *EGFR*-T790M and *HER2*-mutated cell lines [25]. After a phase I trial, recruiting pretreated NSCLC patients and establishing the MTD at 45 mg orally daily [26], a phase II study, performed in patients with NSCLC after failure of ≥ 1 chemotherapy regimen and erlotinib, showed a promising activity and a meaningful improvements in the patient-reported outcomes (PROs) [27]. A large phase II study randomly assigned 188 pretreatred NSCLC to dacomitinb or erlotinib. In the overall population PFS, the primary end-point of the study was 12.4 weeks for dacomitinib arm and 8.3 weeks for erlotinib arm; the PFS benefit was consistent across several subgroups and particularly remarkable in patients with *KRAS* wild-type tumors, with a median PFS of 16.1 weeks versus 8.3 weeks in the experimental and control arm respectively [28]. The ongoing ARCHER 1009 study, a phase III, multicenter, double-blinded trial comparing dacomitinib to erlotinib in pretreatred NSCLC will clarify whether new generation EGFR-TKIs are superior to first-generation agents particularly in the *KRAS* wild-type population [29]. A recent study evaluated the efficacy of dacomitinib in front-line setting in NSCLC patients with activating EGFR mutations (Table 2). The study showed that dacomitinib is particularly effective with a response rate of 74% and a median PFS of 16.8 months [30]. Based on these data, the ARCHER 1050, a phase III, open label trial has been designed to compare the efficacy of first line dacomitinib versus gefitinib in 440 *EGFR* mutant patients with stage IIIB/IV NSCLC. The primary endpoint is PFS by Independent Radiologic Review. This trial is ongoing, with no data currently available [31].

**Table 2:** **First line trials of 2** ^**nd**^ **generation TKI in EGFR mutated patients**

	N	OR%	mPFS (mo)	mOS (mo)
**Dacomitinib**				
Phase II trial^30^	92	74	17	NR
**Afatinib**				
**LUX LUNG II trial** ^**34**^				
*All EGFR mutations*	129	61	10.1	24.8
*Exon 19*–*21 mutations*	106	66	13.7	38.7
**LUX LUNG III trial** ^**35**^				
*All EGFR mutations*				
Afatinib	230	56	11.1	NR
Cis/Gem	115	23	6.9	NR
*Exon 19*–*21 mutations*				
Afatinib	204	-	13.6	NR
Cis/Gem	104	-	6.9	NR
**LUX LUNG VI trial** ^**39**^				
*All EGFR mutations*				
Afatinib	242	66.9	11.1	
Cis/Gem	122	23	5.6	
**XL647**				
Phase II trial^53^				
*All EGFR mutations*	41	20	5.3	19
*Exon 19*–*21 mutations*	14	57	9.3	NR

### Afatinib

Afatinib is an orally, irreversible EGFR, HER2 and HER4 inhibitor, showing preclinical activity against cancer cells harboring common activating *EGFR* mutations and the *T790M* mutation, albeit with a lower potency [32]. Phase 1 studies established the MTD at 50 mg orally daily, with diarrhea and rash as most common adverse event [33]. The role of afatinib in first line setting has been investigated in three studies (Table 2). The first, the LUX-LUNG 2 trial, was a phase 2 trial exploring the efficacy of afatinib in patients with stage IIIb/IV, *EGFR* mutated NSCLC. The study, enrolling patients untreated or previously exposed to chemotherapy, was subsequently amended to allow only untreated patients. The primary endpoint was objective response rate by Independent Radiologic Review. Among the 129 enrolled patients, 61 received afatinib as first-line treatment and 68 in second line, 99 received 50 mg orally daily as starting dose and 30 received 40 mg orally daily; 106 patients presented the common exon 19 or 21 *EGFR* mutations and 23 the other less common mutations. The objective response rate in the overall population was 61% by independent review and 60% by investigator assessment. In the *EGFR*-mutated patients, the objective response rate by independent review was 66%, whereas was 39% in patients with uncommon *EGFR* mutations. Drug-related adverse event, mainly diarrhea and skin rush were observed in the vast majority of cases, with about a quarter of patients experiencing grade 3 adverse events with 50 mg dose. These data indicated that the daily dose of 40 mg was preferable for additional studies [34]. Two other phase III trials, in the same setting, have been subsequently designed. The LUX-LUNG 3 study [35], a multicenter, randomized, open-label phase III study compared afatinib with cisplatin plus pemetrexed in patients with lung adenocarcinoma, stage IIIb/IV harboring *EGFR* mutations [36, 37]. Among the 1,269 screened patients, 345 resulted eligible and were randomized in a two-to-one fashion to afatinib 40 mg daily or chemotherapy up to a maximum of six cycles (without any maintenance therapy). As expected, patients were mainly East Asian, never-smokers and women; *EGFR* mutations were predominantly exon 19 deletions and L858R point mutations. The PFS assessed by independent review (primary endpoint) has been significantly prolonged in the afatinib arm compared to chemotherapy arm, with median PFS of 11.1 and 6.9 months, and 13.6 versus 6.9 months in patients with classical (exon 19 deletion or exon 21) *EGFR* mutations. Afatinib has achieved a higher response rates compared with chemotherapy according to both independent (56% versus 23%) and investigator (69% versus 44%) assessment, and higher disease control rate (90% versus 81% by independent review). The most frequent (≥20% incidence) adverse reactions from afatinib were diarrhea, rash/dermatitis acneiform, stomatitis, paronychia, dry skin, decreased appetite and pruritus; treatment-related adverse events grade ≥ 3 occurred in 112 patients (49%) receiving afatinib but therapy was discontinued just in 8%; predose plasma samples on days 1 and 8 of cycle two and day 1 of cycle three demonstrate that dose modification, due to individual tolerability, optimized the exposure to afatinib, holding efficacious plasma levels [38]. In the third study, the LUX LUNG 6 [39], Asian patients harbouring*EGFR* mutations have been randomized in a two-to-one fashion to afatinib 40 mg daily or cisplatin plus gemcitabine. The study showed that patients treated with afatinib had a significantly longer PFS than individuals receiving chemotherapy (median PFS 11.0 versus 5.6 months, p < 0.0001), as well as higher response rate (66.9% versus 23.0%, p < 0.0001) and higher disease control rate (92.6% versus 60.2%, p < 0.0001).

Two prospective studies have investigated the role of afatinib in patients with acquired resistance to first generation EGFR-TKIs (Table 3). The first reported trial, the LUX LUNG 1 [40, 41], randomly allocated 585 NSCLC patients to afatinib 50 mg daily or placebo. Eligible patients had received one or two previous chemotherapy regimens and had disease progression after at least 12 weeks of treatment with erlotinib or gefitinib. The median OS (primary endpoint) was not different even if a significant difference in PFS was observed (3.3 versus 1.1 months, p < 0.0001 by independent review). In the same setting, the LUX LUNG 4 trial [42] enrolled 62 Japanese patients showing 8.2% response rate and a median PFS of only 4.4 months. Overall these data indicate that afatinib is modestly effective in patients with EGFR-TKI acquired resistance and the best setting for using the agent is in front-line only in patients harbouring *EGFR* mutations.

**Table 3:** **Trials of 2** ^**nd**^ **generation TKI in acquired resistance setting**

	N	OR%	mPFS (mo)	mOS (mo)
**Afatinib**				
**LUX LUNG I trial** ^**40**^				
Afatinib	390	7	3.3	10.8
Placebo	195	<1	1.1	12.0
**LUX LUNG IV trial** ^**42**^	61	8.2	4.4	19
**XL647**				
Phase II trial^52^	41	3	3.6	16.1

<, less than.

In EGFR-TKI pretreatred patients, preclinical models suggested that combination of afatinib with cetuximab, a monoclonal antibody against the extracellular domain of the EGFR, is particularly effective in presence of acquired resistance [43].The superiority of the combination is hypothesized to be due to cetuximab’s ability to cause down-regulation of EGFR and afatinib’s ability to block its kinase activity, leading to significantly lower level EGFR pathway signaling, even with a T790M mutation. Based on these findings, a large phase Ib/II study showed a promising 32% response rate among the 53 individuals with EGFR-T790M mutation [44, 45]. Although cetuximab-afatinib combination showed promising results, current data do not justify its use outside clinical trials.

### Targeting a specific alteration of the EGFR downstreamingsignalling

Recently, investigators have identified covalent pyrimidine EGFR inhibitors, which are 30–100 fold more potent than quinazoline based EGFR inhibitors against *EGFR* T790M cells, and up to 100 fold less potent against wild type EGFR cells, as CO-1686 and AP26113 [46].

CO-1686, an oral covalent TKI, has been investigated in a phase I/II study [47], enrolling T790M-mutated patients pretreated with first generation TKIs. Preliminary results suggested a relevant activity and satisfactory tolerability, with the absence of typical adverse events derived from EGFR inhibition. AP26113, a novel TKI that potently inhibits mutant activated forms of ALK, EGFR and TKI-resistant forms including T790M positive, is nowadays testing in a phase I dose finding study [48] with initial evidence of activity in *EGFR* mutated patients progressed to prior TKI therapy.

Considering the high selective target, this class of inhibitors could likely represent a real chance of treatment for patients with EGFR-TKI acquired resistance, after the necessary confirmation derived from large, prospective, randomized trials.

### Combinating the EGFR plus alternate pathways inhibition

Vascular endothelial growth factor (VEGF) pathway has been extensively studied as therapeutic target in NSCLC. Several studies demonstrated the existence of a cross-talk between VEGF and EGFR pathways, being the EGFR a regulator of VEGF expression, in turn associated with resistance to EGFR inhibition [49, 50]. Therefore, there is a strong rationale for using strategies inhibiting both targets as the multi-targeted TKI agents XL647 and BMS-690514.

XL647, an oral small-molecule inhibitor of EGFR, VEGFR-2, HER2 and Ephrin type-B receptor 4 (EphB4), in preclinical studies showed efficacy against *EGFR*-driven tumors, including those harboring *T790M*[51]. However, a phase II trial enrolling patients with EGFR-TKI acquired resistance, demonstrated a disappointing 3% RR, not supporting further investigation [52], Table 3. At the same time, another phase II trial of first-line XL647 in a population enriched for *EGFR* mutations (i.e. lung adenocarcinoma, never-smokers, females, *EGFR* mutated patients) (Table 2) showed a RR of 19.6% in the overall population and a RR of 57% among the 14 patients harbouring an activating *EGFR* mutation. These data suggested that the efficacy of XL647 is confined to the *EGFR* mutated population [53]. In a subsequent exploratory study, Chmielecki et al. investigated the clinical characteristics of eight patients with metastatic EGFR-mutant lung adenocarcinoma who were treated first-line with XL647 and then progressed. The study showed that, among the 5 tumor samples collected at the time of XL647 failure, only one harbored the T790M mutation and that three patients treated with second line erlotinib derived additional long-term benefit from the EGFR TKI [54]. Overall, the study emphasize the potential role of XL647 as an agent that do not necessarily select for T790M-mediated resistance and allow the sequential use of non-cross-resistant EGFR TKIs.

BMS-690514, a reversible oral inhibitor of EGFR, HER-2 and −4, VEGFRs-1 to −3, showed antitumour activity in tumour xenograft models and in cell lines containing the EGFR T790M mutation, suggesting a role against erlotinib-resistant tumours [55]. A phase I/II trial has recently evidenced a modest activity and disease control in both erlotinib-naıve and erlotinib-resistant NSCLC patients [56]. A randomised phase II trial comparing BMS 690514 with erlotinib is currently ongoing.

## Conclusion

In the last years, the treatment of NSCLC has dramatically changed. Currently, the treatment’s choice is based on a careful assessment of tumor histology and, most importantly, of biological characteristics, specifically of the *EGFR* and *ALK* status. *EGFR* mutations identify a distinct subgroup of NSCLC which benefit from a first line treatment with first generation EGFR-TKIs, as clearly evidenced by several phase III trials. Second generation TKIs are new agents currently under investigation with the intent to improve the efficacy in the first line setting and to provide a valid treatment option in the acquired resistance setting. New inhibitors such as dacomitinib and afatinib differ from first generation EGFR-TKIs because they form irreversible link to the EGFR kinase domain and inhibit other receptors (i.e. HER2 and HER4). The potential superiority of dacomitinib versus erlotinib in KRAS wild-type NSCLC is currently under investigation in a large phase III trial. Afatinib demonstrated superiority versus standard chemotherapy in two phase III studies and based on the results of the LUX LUNG 3 trial, Food and Drug Administration has recently approved the drug for the first-line treatment of patients with NSCLC whose tumors harbored EGFR exon 19 deletions or exon 21 (L858R) substitution mutations as detected by an FDA-approved test, specifically the *therascreen* EGFR RGQ PCR Kit (QIAGEN). Subsequently, the European Committee for Medicinal Products for Human Use (CHMP) adopted a positive opinion, recommending the granting of a marketing authorisation for afatinib.

From the clinical point of view there are many questions about new EGFR-TKIs including efficacy and toxicity over erlotinib or gefitinib. At the present time there is no phase III study directly comparing new versus old EGFR-TKIs. Based on indirect comparison it seems that new generation EGFR-TKIs produce a similar response rate to erlotinib or gefitinib with a possible improvement in terms of progression-free survival and with an increased risk of side effects particularly skin rash and diarrhea. Available data with afatinib in NSCLC with acquired resistance to erlotinib or gefitinib indicate that this agent is modestly effective in such setting and new drugs and new strategies are urgently needed.

### Disclosure of potential conflicts of interest

The authors have no other relevant affiliations or financial involvement with any organization or entity with a financial interest in or financial conflict with the subject matter or materials discussed in the manuscript. No writing assistance was utilized in the production of this manuscript.
